# Letter to the editor regarding: “A rare clinical presentation of metronidazole-induced dysarthria: A Case report with literature review”

**DOI:** 10.1016/j.radcr.2025.06.025

**Published:** 2025-07-05

**Authors:** Erin Jensen

**Affiliations:** Moderator of the Metronidazole (Flagyl) Toxicity Support Group (online)

*To the Editor*,

With respect to the case report, published by Mitra et al. in May 2025 for *Radiology Case Reports*, Volume 20, Issue 5, there are some concerns with the terminology utilized and conclusions offered for this report I’d like to address. While the publication’s intent is appreciated, as it adds further awareness to metronidazole’s potential adverse effects, I would like you to consider the following revisions to promote better accuracy for the information provided.

Firstly, the case report emphasizes multiple times that metronidazole toxicity typically occurs with “prolonged use or high cumulative doses [1],” which is inaccurate. As stated within the case report, “Dysarthria, characterized by slurred or unclear speech, is an uncommon complication of metronidazole, which is generally well-tolerated but has the potential to cause neurotoxic effects in some individuals, especially with prolonged use or high cumulative doses [1].” The concept of toxicity occurring *especially* with prolonged use or high doses is further emphasized in the case report, “This highlights the importance of considering drug-induced neurotoxicity in patients presenting with new neurological symptoms, particularly when there is a history of prolonged antibiotic therapy,” and lastly, “This case underscores the necessity of closely monitoring patients on metronidazole, especially those receiving prolonged treatment, for any emerging neurological signs [1].”

The concept that metronidazole toxicity occurs mainly with “prologue use or high cumulative doses,” was debunked by the 2011 systematic review, “Metronidazole-induced central nervous system toxicity: a systematic review,” which analyzed the published case reports of 64 patients diagnosed with metronidazole-induced central nervous system toxicity collected from 1965 to 2011 [2].

The conclusion of this analysis is as stated: “Metronidazole can rarely cause central nervous system toxicity; it does not seem to be a dose- or duration-related phenomenon [2].” As the moderator of a metronidazole toxicity support group with approximately 5000 members, we conducted a survey in regards to this topic, asking members about the timeline of their initial adverse reaction to metronidazole, and toxicity occurs within the first 2 weeks of metronidazole ingestion in 94% of group members who responded.

Thus, data from the systematic review combined with patient testimonies strongly support that dysarthria and other signs of neurotoxicity following metronidazole can occur regardless of dosage or duration of use.

Secondly, in the May 2025 case report, it states that metronidazole toxicity is “rare” 6 times [1]. Dysarthria is a less common symptom of metronidazole toxicity; however, stating that it is “rare” lacks objective context leading to subjective deductions. Furthermore, for the final conclusion, “While metronidazole is generally considered safe, awareness among clinicians about these rare adverse effects is crucial for early recognition and management [1],” a more appropriate terminology would be “uncommon,” as, overall, metronidazole toxicity is not rare.

In the article, “Metronidazole-associated Neurologic Events: A Nested Case-control Study,” it was concluded that metronidazole-induced neurotoxicity and/or neuropathy that requires hospitalization, occurs in approximately 1 in 400 patients (0.25%) who have ingested metronidazole within 100 days. Within the article itself, it emphasizes the data’s limitations, as the records obtained were restricted to patients over the age of 66 and only those hospitalized with, “cerebellar dysfunction, encephalopathy, or peripheral neuropathy,” within the 100 days indicated. The article concludes that the authors expect they “undercaptured” the neurologic outcomes [3].

Based on another survey in our metronidazole toxicity support group, 88% of patients who self-reported having symptoms of metronidazole neurotoxicity and/or neuropathy were never hospitalized, and therefore, would not have been included within this article’s collective data. Anecdotally, as a metronidazole toxicity survivor, I was hospitalized after I lost the ability to walk and speak in 2015 while ingesting metronidazole, but was not diagnosed with cerebellar dysfunction until 9 months after my first initial reaction. Therefore, even without the age restriction noted in the article itself, my case would also not have fallen within the parameters of the conclusions presented, further highlighting how much of an “undercapture,” 1 in 400 patients is within the article’s conclusions.

I wish to thank the researchers for taking the time to publish this article. Raising awareness of metronidazole’s potentially dangerous adverse effects is something patients like myself and the others within our support group greatly appreciate. However, there seems to be a piggyback effect of misinformation within case reports like this, using well-meaning but outdated data and terminology that could hinder appropriate diagnosis of patients with acute metronidazole toxicity, which is far more common than typically reported in medical literature.
